# COVID‐19 as a trigger of Guillain‐Barré syndrome: A review of the molecular mechanism

**DOI:** 10.1002/iid3.875

**Published:** 2023-05-16

**Authors:** Mahdi Malekpour, Shaghayegh Khanmohammadi, Mohammad Javad Entezari Meybodi, Dorsa Shekouh, Mohammad Reza Rahmanian, Sina Kardeh, Negar Azarpira

**Affiliations:** ^1^ Student Research Committee Shiraz University of Medical Sciences Shiraz Iran; ^2^ Transplant Research Center Shiraz University of Medical Sciences Shiraz Iran; ^3^ Network of Immunity in Infection, Malignancy and Autoimmunity (NIIMA) Universal Scientific Education and Research Network (USERN) Tehran Iran; ^4^ Research Center for Immunodeficiencies, Children's Medical Center Tehran University of Medical Sciences Tehran Iran; ^5^ Central Clinical School Monash University Melbourne Australia

**Keywords:** COVID‐19, GBS, Guillain‐Barré syndrome, long COVID, postacute COVID‐19 syndrome, SARS‐CoV‐2

## Abstract

Severe acute respiratory syndrome coronavirus 2 (SARS‐CoV‐2) caused a pandemic with serious complications. After coronavirus disease 2019 (COVID‐19), several post‐acute COVID‐19 syndromes (PACSs) and long‐COVID sequels were reported. PACSs involve many organs, including the nervous, gustatory, and immune systems. One of the PACSs after SARS‐CoV‐2 infection and vaccination is Guillain‐Barré syndrome (GBS). The incidence rate of GBS after SARS‐CoV‐2 infection or vaccination is low. However, the high prevalence of COVID‐19 and severe complications of GBS, for example, autonomic dysfunction and respiratory failure, highlight the importance of post‐COVID‐19 GBS. It is while patients with simultaneous COVID‐19 and GBS seem to have higher admission rates to the intensive care unit, and demyelination is more aggressive in post‐COVID‐19 GBS patients. SARS‐CoV‐2 can trigger GBS via several pathways like direct neurotropism and neurovirulence, microvascular dysfunction and oxidative stress, immune system disruption, molecular mimicry, and autoantibody production. Although there are few molecular studies on the molecular and cellular mechanisms of GBS occurrence after SARS‐CoV‐2 infection and vaccination, we aimed to discuss the possible pathomechanism of post‐COVID‐19 GBS by gathering the most recent molecular evidence.

## Graphical abstract

Due to the importance of Guillain−Barre syndrome as a long‐COVID disease, this review performs information about the molecular mechanism of post‐acute severe acute respiratory syndrome coronavirus 2.

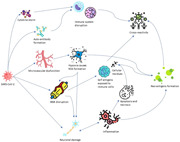

## 1 INTRODUCTION

Severe acute respiratory syndrome coronavirus 2 (SARS‐CoV‐2), a new type of Coronaviridae family,^1^ emerged in Wuhan, China, in December 2019.^2^ The coronavirus disease 2019 (COVID‐19) pandemic drastically affected human life. World health organization (WHO) estimated a mortality rate of 3.4% at the early stages of the COVID‐19 pandemic.^3^ Based on the United States of America simulation, studies estimated the economic burden of COVID‐19 as about 163.4 billion dollars only for medical costs.^4^ High mortality rates, treatment costs, lockdowns, and many other factors have affected the economy and healthcare systems of many countries worldwide.^5^

Coronaviruses are enveloped, positive‐sense, single‐stranded RNA and cause infection in many host species.^6^ Along with lung involvement, SARS‐CoV‐2 infection affects many organs, including the kidneys, liver, heart, and brain.^7^ During SARS‐CoV‐2 infection, neurological involvements, such as olfactory and gustatory impairments, are also common. Patients with COVID‐19 can present neurological symptoms due to either viral neurological damage or indirect neuroinflammatory and autoimmune processes.^8^ Also, patients have prolonged neurological complications following COVID‐19, known as long COVID complications.^9^ Guillain−Barré syndrome (GBS), bell's palsy, and hemorrhagic stroke after COVID‐19 and vaccination have been reported among their complications.^10^, ^11^

GBS is the most frequent acute peripheral neuropathy, characterized by numbness, progressive weakness, pain, paralysis, tingling, and autonomic dysfunction.^12^, ^13^ Like many other diseases, both genetic and environmental factors play an important role in the occurrence of GBS.^14^ The worldwide estimation of the GBS incidence is between 1.1/100,000 and 1.8/100,000 persons per year and increases with age.^15^, ^16^ Based on serological data, the incidence of GBS in patients with COVID‐19 is 1 per 62,500 persons^17^; this incidence rate is lower than other infections that are known to have an association with GBS, like the Zika virus (∼1 in 4,000) and *Campylobacter jejuni* (∼1 in 1000).^18^, ^19^, ^20^ It is notable that during the COVID‐19 pandemic, factors such as lockdown periods affect the prevalence of GBS worldwide, resulting in different statistics from different countries.^21^, ^22^ However, severe complications of GBS increase the importance of GBS occurrence after COVID‐19. On the other hand, reports of the occurrence of GBS after COVID‐19 vaccination increase the importance of this possible association.^23^, ^24^

Respiratory failure occurs in 20‐30% of GBS patients and, together with autonomic dysfunction, accounts for 3‐10% of the mortality rate of GBS.^13^, ^25^, ^26^ Despite intravenous immunoglobulin (IVIG) therapy and plasmapheresis, up to 20% of patients are severely disabled and develop lifelong problems after 6 months.^26^, ^27^ The recurrence rate of GBS is estimated to be 1%–6%.^28^ Given the importance of GBS after COVID‐19, we aimed to determine the association of GBS with SARS‐CoV‐2 infection and vaccination by explaining the possible pathomechanism of GBS and SARS‐CoV‐2.

## 2 OVERVIEW OF SARS‐COV‐2 EFFECTS AND GBS PATHOGENESIS

### 2.1 Effects of SARS‐CoV‐2 on the neurological system

Our understanding of COVID‐19 has changed from a naive conjecture of severe respiratory syndrome to a complex disease with multiorgan complications.^29^ Established literature on COVID‐19 reported many complications, including systemic pulmonary, cardiovascular, digestive, hematologic, neurocognitive, and immunologic complications.^29^, ^30^

Neurocognitive complications in patients with COVID‐19 are common in hospitalized, partially recovered, and post‐acute COVID‐19 syndrome (PACS) patients. More than 80% of hospitalized patients and up to 35% of PACS patients may have neurologic symptoms during their diseases.^31^ The common neuro‐complications of COVID‐19 are summarized in Table 1.

**Table 1:** The common neuro‐complications of COVID‐19 during and after COVID‐19 infection.

Acute COVID‐19 complication	Percentage^32^	Post‐acute COVID‐19 complication	Percentage^33^
Gustatory dysfunctions	38.5	Fatigue	36.75
Olfactory dysfunctions (hyposmia/anosmia)	35.8	Brain fog	32.17
Myalgia	19.3	Sleep disturbance	30.67
Headache	14.7	Memory issue	28.04
Altered mental status	9.4	Anxiety	22.97
Dizziness	8.77	Attention disorder	21.84
Nausea and vomiting	4.6	Myalgia	17.22
Neuralgia	2.3	Headache	15.33
Ataxia	0.3	Olfactory dysfunctions (hyposmia/anosmia)	11.99

Rapid viral replication, direct cell damage, mitochondrial pathway dysregulation, immune system activation, and inflammatory mediators are the main causes of the acute symptoms and long‐term sequelae of SARS‐CoV‐2 infection, such as cognitive, neurodegenerative, and demyelinating disorders.^30^

### 2.2 GBS pathogenesis

GBS is caused by an impaired immune system that damages peripheral nerve tissue. Attacking the nerve cells can be triggered by infection, surgery, or immunization.^34^ Cross‐reactivity between pathogen antigens and nerve tissue is the leading cause of GBS.^35^ One‐quarter of patients with GBS have had a history of a recent infection with *C. jejuni*.^36^ Also, newly emerged pathogens like the Zika virus, enterovirus D68, and SARS‐CoV‐2 can cause GBS.^35^, ^37^ Studies on COVID‐19‐related GBS commonly reported sensorimotor demyelinating GBS with frequent facial palsy.^38^ Patients with simultaneous COVID‐19 and GBS seem to have higher admission rates to the intensive care unit (ICU).^24^ Higher rates of admission to the ICU can be due to the simultaneous respiratory problems caused by GBS and COVID‐19. Also, demyelination was more aggressive in post‐COVID‐19 GBS patients than in other GBS patients.^24^ However, IVIG therapy in GBS patients of PACS showed promising effects.^39^

## 3 POST‐COVID‐19 GBS PATHOGENESIS

### 3.1 Neurotropism and neurovirulence of SARS‐CoV‐2

SARS‐CoV‐2's neurotropism and capacity to infect nerve cells have been demonstrated earlier.^40^ The neurotropism of SARS‐CoV‐2 adds it to a list of neuro‐invasive coronaviruses, such as the Middle East respiratory syndrome coronavirus and SARS‐CoV.^41^, ^42^

Coronaviruses can infect different cell types, including epithelial cells in the respiratory tract, gastrointestinal (GI) tract, and kidney.^43^ Two primary processes are involved in mediating viral entry into the host cell. The first stage is the attachment of the viral spike (S) protein, a class I fusion protein, to the host receptor on the cell membrane, followed by the fusing of the plasma membranes, which is facilitated by the angiotensin‐converting enzyme 2 (ACE2) receptor and transmembrane serine protease 2 (TMPRSS2). Other host cell receptors, such as Basigin (BSG; CD147) and Neuropilin‐1 (NRP1), can also allow the entrance of the virus via the spike.^44^ The viral envelope membrane fuses with the host endosome or lysosome in the second stage. Cathepsin L, which may also compensate for the role of TMPRSS2 in cells lacking TMPRSS2, facilitates this membrane fusion process.^45^

SARS‐CoV‐2 can reach the central nervous system (CNS) directly and indirectly. The virus can invade the brain via the olfactory nerve and then spread through trans‐synaptic transport. Additionally, the virus can pass the blood−brain barrier (BBB) to enter the CNS by migrating through infected leukocytes or after infecting and damaging capillary endothelial cells. Other possible entry routes include infection of peripheral nerves, which might lead to retrograde axonal transport of virus particles into the CNS and infection of the vagus nerve and the GI tract.^46^, ^47^ Once the virus enters the brain tissue, it can interact with neurons and non‐neuronal cells, primarily astrocytes, oligodendrocytes, and endothelial cells, which express ACE2 receptors and are distributed across the brain. The large spectrum of neurological complications associated with COVID‐19 infection can partially be explained by this distribution.^48^ Although limited studies have shown the viral particles in the cerebral spinal fluid (CSF) of COVID‐19 patients, the co‐occurrence of some GBS cases during COVID‐19 increased the probability of the neuro‐invasive effects of COVID‐19 on the peripheral nervous system (PNS).^49^

After infection, the virus can cause an extensive, systemic immune response that results in uncontrolled, ongoing inflammation known to cause various neurological manifestations. This may result in neurological barrier disruption and the subsequent increase in the inflammatory markers and reactive oxygen species (ROS) in the brain, linked to dysfunction and neuron damage. Furthermore, it is known that the release of interleukin‐6 (IL‐6) and high d‐dimer levels increase vascular permeability and induce complement and coagulation cascades, which in turn cause various acute cerebrovascular events such as stroke.^50^, ^51^, ^52^ A localized proinflammatory environment can result from the immune system's prolonged and hyperactive response to a viral infection, which sequesters and exposes auto‐antigens to the immune system. Immunoglobulins (IGs) or immune complexes have the potential to cause neuronal injury by triggering proinflammatory reactions in neural tissues.^53^ Autoimmune reactions can trigger the neurodegeneration process through autoantibody‐mediated mechanisms. Increased immune‐mediated damage, like that observed in myasthenia gravis and multiple sclerosis (MS), can result from the production of inflammatory markers.^54^

### 3.2 Microvascular dysfunction

During the SARS‐CoV‐2 pandemic, several vascular and thrombotic events were reported worldwide.^55^ On the other hand, studies showed capillary dysfunction in moderate to severe patients with COVID‐19.^56^ Many long‐COVID symptoms, such as fatigue and chest pain, are related to long‐lasting microcapillary disruption.^57^ SARS‐CoV‐2 can disrupt microvascular functions directly and indirectly by reducing nitric oxide bioavailability, oxidative stress, direct virulence, and autoantibodies.^58^, ^59^ Microvascular disruption can induce thrombotic events like myocardial infarction after COVID‐19.^58^, ^60^ Microvascular changes can cause prolonged hypoxia and acidosis, resulting in blood‐nerve barrier (BNB) damage and neuropathies, such as diabetic neuropathy. Many reports of GBS occurrence after diabetic neuropathy suggest that GBS is associated with vascular changes.^61^, ^62^, ^63^, ^64^ Hypothetically, the disruption of microvascular function during SARS‐CoV‐2 infection can contribute to the occurrence of GBS; further studies are needed to confirm this phenomenon.

### 3.3 BNB disruption

The BNB supplies an essential endoneurial microenvironment for peripheral nerves to maintain homeostasis.^65^ Tight junctions can maintain endoneurial homeostasis by forming a barrier against endoneurial microvessels. Tight junction protein complexes, including claudins, seal the barriers and control ion, water, and cell influx and efflux between the bloodstream and endoneurium.^66^ BNB also prevents interactions between immune cells and nerve cells as well as damage to neurons by preventing the collision of inflammatory substances and free radicals during infection or systemic inflammation.^67^, ^68^

Many inflammatory neuropathies start in the absence of BNB, like the involvement of neuromuscular junction in Miller‐Fisher Syndrome (MFS), a subtype of GBS. Damage to the BNB and, consequently, to the neurons can stimulate immune cells, and the lack of suppression of the immune system, especially in inflammatory conditions, can cause GBS.^68^, ^69^, ^70^ One of the main hypotheses is that after BNB disruption, neural cells are exposed to circulating autoantibodies.^71^ Barrier disruption with consequent edema is an early key component leading to autoimmune polyneuropathies, such as GBS.^72^ ACE2 receptors are expressed at high levels in endothelial cells, neurons, and glial cells, which endorse speculation about direct cytopathogenic effects. The expression of the ACE2 receptor on glial cells can lead to direct BNB neurovirulence causing the BNB disruption as seen previously in BBB disruptions by COVID‐19.^38^, ^73^ Since most of the COVID‐19‐related GBS cases reported a demyelinating variant of GBS^38^ and antibodies have a significant impact on the COVID‐19 pathogenesis,^74^ it can be anticipated that antibodies may have a critical role in post‐COVID GBS pathogenesis. However, anti‐ganglioside antibodies, the main auto‐antibodies against myelin that cause GBS, are more frequent in axonal forms of GBS.^75^ Antiganglioside antibodies are low in post‐COVID‐19 GBS patients. Thus, the spectrum of the immune cascade should be expanded by studying other different antibodies affecting the myelin sheath, Schwann cell components, the neuronal axolemma, and, generally, BNB function.^38^

It was found that incubation of bovine cell lines with GBS sera leads to disruption of the endoneurial barrier. It is not fully understood whether antibodies directly cause the barrier breakdown or whether opening via inflammatory factors elicits changes and ultimately causes demyelination.^76^ Recent evidence suggests that immune cells elevate the permeability of the BNB during viral infections.^65^ In viral infections, interferon‐γ (IFN‐γ) locally secreted by virus‐specific CD4 T cells increased the permeability of the BNB, enabling circulating antiviral antibodies to defend against the viruses.^77^ On the other hand, a pathologic hallmark of several inflammatory peripheral neuropathies is the infiltration of hematogenous leukocyte subpopulations in peripheral nerves and nerve roots, as observed in situ on well‐defined nerve biopsies.^78^ Acute inflammatory demyelinating polyradiculoneuropathy (AIDP), another subtype of GBS, is also characterized pathologically by macrophage‐mediated demyelination, predominantly monocytes, and less commonly T and B lymphocyte infiltration into the peripheral nerve and nerve root endoneurium.^78^ The mechanisms of leukocyte infiltration across the BNB highlighted CD11b‐mediated leukocyte trafficking. CD11b is the integrin responsible for mononuclear leukocyte adhesion, with monocytes/macrophages being the most prevalent leukocyte subpopulation trafficking at the BNB.^79^ CD11b+ leukocytes directly interacted with endoneurial microvessels as well as endoneurial sites. CD11b+ leukocytes are associated with disrupted Schwann cell membrane organization.^80^, ^81^

### 3.4 Hyperactive immune system and GBS

Growing evidence indicated that SARS‐CoV‐2 triggers immune‐mediated neurological consequences. The exact pathophysiological mechanism is still unknown, but there are some shreds of evidence of potential mechanisms. Failure to detect the virus in the CSF mainly supports the immune‐mediated mechanism of neurological complications instead of the direct effects of the virus.^82^ Lucchese and Flöel hypothesized that SARS‐CoV‐2 infection triggers the adaptive system, and the interactions of T‐cell and B‐cell trigger the production of antibodies, specifically mimicking the ganglioside or peptide structures or sequences, disrupting self‐tolerance.^82^ Self‐tolerance disrupts when a clonal lineage of lymphocytes reacts to self‐antigens and cannot become inactivated.^83^

“Cytokine storm” triggered by SARS‐CoV‐2 is another proposed mechanism of the autoimmune pathway that possibly could result in COVID‐19‐associated GBS. The exponential increase in cytokines, including IFN‐γ, tumor necrosis factor‐α (TNF‐α), IL‐1β, IL‐6, and IL‐17, besides other chemokines, has been observed, which could induce multiple organ damage. Especially, IL‐6 could be a promising factor for detecting the severity of the disease,^84^ and there is a shred of evidence indicating how anti‐IL‐6 could be effective in critically ill cases of COVID‐19.^85^ Consequently, macrophage activation syndrome (MAS) could happen following the cytokine storm initiating the critical sequence of COVID‐19. Indeed, macrophage activation induces the overproduction of cytotoxic proinflammatory cytokines.^86^ Following the immune system dysregulation or MAS/secondary hemophagocytic lymphohistiocytosis, the overproduction of IL‐6, CD4 lymphopenia, and B cell lymphopenia is triggered. These changes could induce autoimmune processes.^87^ Simultaneously, the exponential multiplication of the virus results in the depletion of the natural killer (NK) cells. The depletion of the NK cells causes severe lung injury and damage to other tissues, including the nervous system; in contrast, the immunosuppressive ability of NK cells could protect the nervous system from acquired GBS.^87^, ^88^

Many studies have indicated the pivotal role of “cytokine storm” in initiating typical GBS.^89^, ^90^ The level of IL‐4, IL‐17, IL‐22, and IFN‐γ detected in CSF are potentially associated with the severity of the disease.^91^ Evidence indicated that the “cytokine storm” increases the permeability of BBB to pathogenic circulating proteins, including the antibodies and other mediators; otherwise, it may allow the immune system to react against protected nervous system antigens. The recent studies mainly indicated the “cytokine storm” pattern of molecules detected in CSF rather than an antibody‐mediated mechanism, mostly glial markers, TNF‐ α, and IL‐6.^92^, ^93^ As described, “cytokine storm” can also affect BNB, theoretically resulting in the reaction of immune cells against protected nervous system antigens, as seen in GBS patients.^94^

SARS‐CoV‐2 can induce a hyperactive immune system.^95^ During the immune response, Neutrophils can form extracellular traps (NETs).^96^ Indeed, inflammation can trigger NET formation, and NETs themselves trigger inflammation.^95^ On the other hand, NETs could serve autoantigens produced during the apoptosis of human cells in the inflammatory state, leading to autoantibody production.^95^ The mentioned mechanism of auto‐antibody production could lead to GBS, which needs more studies for confirmation.

### 3.5 Molecular mimicry and autoantibodies

Auto‐antibodies have an essential role in the pathogenesis of COVID‐19. Autoantibodies have been detected in about 10%−15% of severe patients with COVID‐19. These autoantibodies can induce thrombosis, disrupting cytokine signaling or neutralizing interferons.^74^ Pascolini et al. detected at least one type of autoantibody in 45% of patients, which was associated with significantly more COVID‐19 complications.^97^ The autoantibodies against interferons can neutralize the interferons, which results in the higher replication of SARS‐CoV‐2 and induce the hyper‐inflammatory state of the body.^98^ As could be concluded, the autoimmunity mechanisms, including auto‐antibodies, have a crucial role in the pathogenesis of COVID‐19 itself.

When the sequence of the human proteins is similar to other antigens, molecular mimicry between these two antigens happens.^99^ Various sequence analyses found similarities between human peptides and SARS‐CoV‐2 proteins.^100^ One of the interesting findings was the similarity between human heat shock proteins (HSPs) 90 and 60 and SARS‐CoV‐2 proteins. The role of the HSPs in autoimmunity processes in GBS had been demonstrated earlier.^99^ Also, another bioinformatic analysis found molecular mimicry between the spike protein of SARS‐CoV‐2 and different neuronal proteins and antigens showing the potentiality of cross‐reactivity.^101^

On the other hand, autoantibodies are one of the main reasons for the occurrence of GBS. The variety in GBS symptoms is due to different affected sites in the nervous system.^102^ Also, in each subtype of GBS, specific autoantibodies are generated, which contributes to the detection of the subtype.^103^, ^104^ Gangliosides are the main antigens on nerve and myelin cells in GBS patients as the targets of autoantibodies.^105^ Gangliosides are similar to bacterial lipopolysaccharides, like lipo‐oligosaccharides of *C. jejuni*.^106^ Anti‐GQ1B antibodies are one of the main autoantibodies of MFS, a subtype of GBS.^103^ A variety of autoantibodies against human antigens, including neurofascins,^107^ sulfatides,^108^ and gangliosides,^109^ were found among the post‐COVID‐19 GBS patients. Previous studies showed that antiganglioside antibodies are present in a few proportions of GBS patients after COVID‐19.^109^

It is also possible that neoantigens are randomly formed during inflammatory processes, primarily due to the presence of ROS and reactive nitrogen species. Due to the enhancement of apoptosis and reduced apoptotic and necrotic cell clearances, highly autoimmunogenic antigens can be formed. These new antigens may be similar to those found within the cells. Because of this similarity, immune cells may also react to the self‐antigens, as seen in other AIDs.^110^, ^111^, ^112^ Therefore, dysregulation in maintaining cellular redox levels may play a significant role in the pathogenesis of neurological disorders, such as GBS, epileptic disorders, and demyelinating dementia.^113^, ^114^ As a result of the inflammatory state in the body, several stimulatory factors are produced in the inflammation site, which increases the risk of self‐antigen detection by immune cells. In some patients, simultaneous presentation of self‐antigens and stimulatory factors could break the tolerance against self‐antigens and consequently lead to the occurrence of AIDs.^115^ As a result, it seems that the mechanism explained for the production of auto‐antibodies could be one of the reasons for the occurrence of GBS after COVID‐19, which needs to be confirmed by further studies.

**Figure 1:**
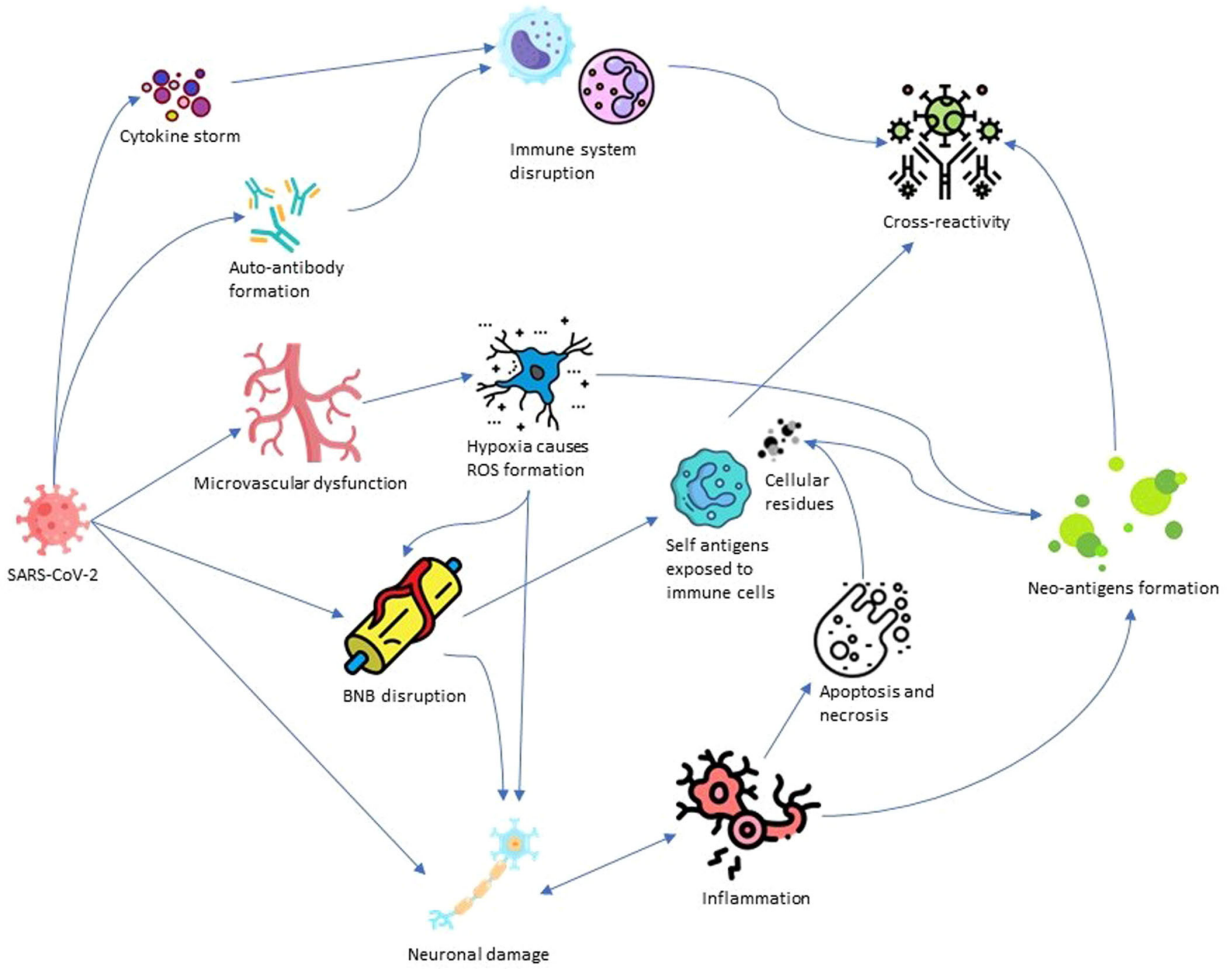
The illustration of the pathomechanism of GBS caused by SARS‐CoV‐2. GBS, Guillain‐Barré syndrome; SARS‐CoV‐2, Severe acute respiratory syndrome coronavirus 2.

Molecular mimicry in GBS pathogenesis can cause the detection of neural antigens by cytotoxic T lymphocytes and the secretion of cytotoxic factors such as perforin, which leads to the damage and destruction of local cells at the site of this collision.^116^ Also, in adaptive immunity, the secretion of antibodies against the self‐antigens activates other immune cells and the complement system.^117^ Activation of the complement system can form the membrane attack complex (MAC) at the surface of myelin and nerve cells, making pores on the cell surface. MAC causes calcium influx into neuronal terminals through complement pores. It results in uncontrolled exocytosis, axonal cytoskeleton degradation, and calcium‐mediated mitochondrial death, accompanied by paralysis.^118^ Besides, complement activation results in voltage‐gated sodium channel disruption. Consequently, these changes can damage the axons, attract the macrophages, and interrupt axons and myelin.^119^, ^120^ Presynaptic Schwann cells of GBS patients may also be damaged due to the injury of nerve terminals and activated complement products, which can cause the vesiculation of myelinated nerve cells.^118^, ^121^ The summarized pathomechanism of GBS caused by SARS‐CoV‐2 can be seen in Figure 1.

## 4 PREDISPOSING FACTORS

### 4.1 Gut dysbiosis

The GI manifestations of SARS‐CoV‐2, like diarrhea and abdominal discomfort, are present in many patients of SARS‐CoV‐2.^122^ On the other hand, infection of SARS‐CoV‐2 could spread to GI cells without symptoms, increasing the frequency of involvement of the gut during the SARS‐CoV‐2 infection.^122^ In hospitalized patients with COVID‐19, gut dysbiosis was seen, lasting even after the SARS‐CoV‐2 resolution.^123^ COVID‐19‐associated gut dysbiosis could correlate with AIDs like GBS by immune system imbalance and bacterial translocation^124^, ^125^ or facilitating secondary bacterial infection.^126^

Gut microbes and the immune system have been co‐evolving together lifelong.^127^, ^128^ A normal gut microbiome could help to balance the immune system and protect the body from autoimmunity by strengthening self‐tolerance.^129^ The commensal microbiota species regulate the balance of Th17/regulatory T cells and inflammatory/anti‐inflammatory cell subsets of the immune system by affecting their development and function.^130^ It is while SARS‐CoV‐2 can fascinate the replacement of the beneficial microbiota with opportunistic species, which persists over time.^131^ A study showed similarities between systemic lupus erythematosus (SLE) patients' gut microbiome and SARS‐CoV‐2. Both diseases can reduce the microbiome's biodiversity, increase pathobionts related to inflammation, and reduce symbionts that are protective against inflammation.^132^ As described before, probiotics could help relieve GBS,^133^ which could be used more widely in patients with COVID‐19.

COVID‐19‐associated gastroenteritis could be another possible trigger for GBS incidence^134^ since the digestive system expresses ACE2, the receptor of SARS‐CoV‐2. Viral nucleocapsid protein, infiltration of lymphocytes in lamina propria, and interstitial edema were seen in patients with COVID‐19.^135^, ^136^ The association of gastroenteritis with GBS has been seen from the past to the present, but its possible mechanism in patients with COVID‐19 needs more investigation.^137^, ^138^

### 4.2 Medications

In the course of hospitalization of patients with COVID‐19, corticosteroids are used to prevent the consequences of septic shock and to reduce the effect of proinflammatory cytokines and “cytokine storm.”^139^ Besides the corticosteroid benefits in COVID‐19 management, overusing corticosteroids in hospitalized patients with COVID‐19 can increase the risk of infections like Mucormycosis.^140^, ^141^ On the other hand, previously has been shown that using immunosuppressive drugs in transplant patients can contribute to the growth of infections like *C. jejuni* in the GI tract and induce GBS.^142^, ^143^ It is known that the antigens of microorganisms, especially *C. jejuni*, can cross‐react with neural antigens, leading to GBS development.^144^, ^145^ Hypothetically, the adequate usage (not over‐usage) of corticosteroids in patients with COVID‐19 could help prevent GBS.

On the other hand, using neurotoxic drugs during the SARS‐CoV‐2 infection, such as hydroxy‐chloroquine, linezolid, ritonavir, and clindamycin, could result in neuropathy after SARS‐CoV‐2 infection.^146^ As previously suggested, different therapeutics could cause GBS.^147^ It could be beneficial to use limited neurotoxic drugs during the SARS‐CoV‐2 infection to prevent GBS occurrence.^147^

### 4.3 Genetic factors

During the COVID‐19 pandemic, different studies showed the effect of genetics on the pathogenic period of the disease, like studies showing the susceptibility of people with different types of human leukocyte antigens (HLAs) to have complicated SARS‐CoV‐2 infections.^148^, ^149^ On the other hand, a study showed a close relationship between the genetics of patients with AIDs, for example, SLE, and the impact of SARS‐CoV‐2 infection on the patients.^150^ Interestingly, a recent case report showed that a patient who had GBS after COVID‐19 shared HLAs with GBS patients. In particular, this patient had HLA‐A33, HLA‐DRB1, and HLA‐DQB1 subtypes, which have been seen before in people who had GBS worldwide.^151^ From this primitive result, the predisposing effect of genetics to GBS after SARS‐CoV‐2 could be concluded.

There are controversial studies about the genetic roots of GBS, but some familial studies showed that genetic patterns could lead to the occurrence of GBS.^14^ Different genes that have specific mutations seem to be related to GBS that contributed to the following three pillars:

1. Genes contributed to identifying, processing, and presenting pathogen antigens.
2. Cytokines and inflammatory factors.
3. Auto‐antigens of neurons.

The first pillar of genes is the cluster of differentiation 1E (*CD1E*), Fc receptor‐like protein 3 (*FCRL3*), Fc gamma receptor (*FcGR*), nucleotide‐binding oligomerization domain‐containing proteins (*NODs*), toll‐like receptor 4 (*TLR4*), and *HLA* subclasses.^14^ HLAs are proteins that help to differentiate between self and non‐self‐antigens and regulate the immune system functions.^152^, ^153^ On the other hand, altered genes that predisposed patients with COVID‐19 to have autoimmunity were suggested to be antigen‐sensing genes, cytokine factors, and lymphocyte activator genes.^98^

Other genes, such as *IL‐10*, *IL‐17*, *TNF‐α*, and *FAS*, are cytokines and factors involved in cytokine signaling and immune system regulation.^14^ Previously was suggested that genetic alteration in cytokines could change the susceptibility to severe SARS‐CoV‐2 infection.^154^ As genetic variations in cytokine genes in GBS patients have been seen before, there could be some associations between these variations and post‐COVID‐19 GBS.

It also appears that defection in the peripheral myelin protein 22 (*PMP22*) gene that encodes surface proteins in nerves can cause GBS.^155^ Due to the importance of the auto‐antigens in the GBS pathogenesis and the foundation of different autoantibodies combinations in SARS‐CoV‐2 induced GBS,^74^, ^109^ there could be genetic variations in the surface proteins of neurons that could increase the susceptibility of patients to GBS. On the other hand, besides the known genetic alterations that contribute to GBS pathogenesis, other genes could predispose patients with COVID‐19 to GBS; further studies are needed to discover the associated genes.

## 5 SARS‐COV‐2 VACCINATION AND GBS

The COVID‐19 pandemic and the idea of achieving herd immunity as a cost‐effective defense mechanism ignited the flame of research for vaccine development. The BNT162b2 (Pfizer–BioNTech), the mRNA1273 (Moderna), the ChAdOx1 nCov‐19 (Oxford–AstraZeneca), and the Ad26.COV2.S (Janssen) vaccines were the first approved vaccines for emergency use.^156^ On the other hand, the idea of an association between GBS and vaccination came from the National Influenza Immunization Program in the USA in 1976, where the incidence of GBS increased after the A/New Jersey influenza vaccination.^157^ Although there are reports of postvaccination GBS with different vaccines,^158^, ^159^, ^160^, ^161^, ^162^ there is little evidence to conclude a causal association between GBS and most vaccines.^163^ A large retrospective study by Chen et al.^164^ did not find evidence of an increased incidence or recurrence of GBS within the 180 days following various disease vaccinations among individuals.^164^ It is while a systematic review documented 73 new cases of GBS and a relapsing episode in 1 patient after COVID‐19 vaccination from case reports and case series.^23^ Several studies show an increased risk of GBS (compared to the expected background rate) after vaccination with adenovirus‐vectored COVID‐19 vaccines (ChAdOx1 nCoV‐19 [Oxford‐AstraZeneca]) and Ad.26.COV2.S (Janssen), but not after mRNA‐based COVID‐19 vaccines (BNT162b2 [Pfizer‐BioNTech] or mRNA‐1273 [Moderna])^11^, ^165^, ^166^, ^167^, ^168^, ^169^, ^170^, ^171^, ^172^; however, Some other studies did not find any significantly increased rate of GBS after COVID‐19 vaccination.^173^, ^174^ However, one of the studies mentions that in some cases, the association between GBS and SARS‐CoV‐2 vaccination should not be perceived as a coincidence.^173^

The exact mechanism of COVID‐19 vaccine‐associated GBS has remained unclear. Molecular mimicry, like what Nachamkin et al.^175^ suggested for the 1976 swine flu vaccine, could be a mechanism for COVID‐19 vaccine‐associated GBS. A study shows molecular mimicry between nucleocapsid phosphoprotein and Orf1ab polyprotein of SARS‐CoV‐2 and HSP90 and HSP60. This molecular mimicry could be responsible for GBS development after SARS‐CoV‐2 infection.^99^ Cross‐reactivity with these antigens could be a mechanism for inactivated virus vaccines, which present the whole virus to the immune system. Kadkhoda^176^ discussed a heptapeptide found in both spike protein of SARS‐Cov‐2 and human neural cell adhesion molecule (NCAM) L1‐like protein, which was proposed as a link between COVID‐19 and GBS.^176^ The casual association between adenovirus‐vectored COVID‐19 vaccines (ChAdOx1 nCoV‐19 [Oxford‐AstraZeneca] and Ad.26.COV2.S [Janssen and Janssen]) and GBS is supported by a study that found an association between adenovirus‐vectored COVID‐19 vaccines and facial paralysis of post‐vaccinated GBS cases.^177^ Almuqrin et al.^178^ detected low levels of viral backbone transcription in the A549 cell line, an immortal cancerous and nonpermissive cell line for vector replication, which could contribute to the autoimmune response.^178^

Kowarz et al.^179^ showed that adenovirus‐vectored COVID‐19 vaccines could lead to the secretion of SARS‐CoV‐2 Spike protein from cells. Adenoviral vectors generate transcripts in the cell nucleus, the place of post‐transcriptional modifications of RNAs like splicing. Loss of the transmembrane anchor of spike protein could happen due to unwanted splicing in adenoviral vectors' transcripts.^179^ The role of SARS‐CoV‐2 spike protein alone or even its S1 subunit in the inflammatory response, endothelial damage, and reducing endothelial barrier function is shown in several studies.^180^, ^181^, ^182^, ^183^, ^184^, ^185^, ^186^, ^187^ Pathologies induced by the S1 subunit could possibly lead to GBS through mechanisms such as BNB disruption and hyperactive immune system, as discussed before.

Despite the increased risk of GBS after vaccination with adenovirus‐vectored COVID‐19 vaccines, GBS occurrence is higher after SARS‐COV‐2 infection than after vaccination.^11^, ^170^ So, vaccination with any COVID‐19 vaccine could decrease the risk of COVID‐19‐induced GBS. However, experts suggest using non‐adenovirus‐vectored vaccines for patients with a history of GBS. If only adenovirus‐vectored vaccines were available, experts would suggest individualized decisions based on the patient's history of GBS and risk for severe SARS‐COV‐2 infection.^188^ Delayed vaccination for patients in the acute phase of GBS, or within 3 months to a year of the onset, and avoidance of vaccination for cases of vaccination‐related GBS (vaccination leads to GBS in a 6‐week window) is also suggested by experts.^189^

## AUTHOR CONTRIBUTIONS

*Conceptualization*: Mahdi Malekpour and Negar Azarpira. *Data gathering*: Mohammad Javad Entezari Meybodi, Dorsa Shekouh, Mohammad Reza Rahmanian, and Sina Kardeh. *Writing*: Mahdi Malekpour, Shaghayegh Khanmohammadi, Mohammad Javad Entezari Meybodi, Dorsa Shekouh, Mohammad Reza Rahmanian, and Sina Kardeh. *Edit*: Mahdi Malekpour, Shaghayegh Khanmohammadi and Negar Azarpira.

## CONFLICT OF INTEREST STATEMENT

The authors declare no conflict of interest.
